# A randomized trial of washed red blood cell and platelet transfusions in adult acute leukemia [ISRCTN76536440]

**DOI:** 10.1186/1471-2326-4-6

**Published:** 2004-12-10

**Authors:** Neil Blumberg, Joanna M Heal, Jacob M Rowe

**Affiliations:** 1 Department of Pathology & Laboratory Medicine (Transfusion Medicine Unit), University of Rochester Medical Center, Box 608, Rochester, NY 14642 USA; 2 Department of Medicine (Hematology-Oncology Division), University of Rochester Medical Center, Box 608, Rochester, NY 14642 USA; 3 Dept. of Hematology and BMT, Rambam Medical Center, Haifa 31096 Israel

## Abstract

**Background:**

Platelet transfusion is universally employed in acute leukemia. Platelet concentrate supernatants contain high concentrations of biologic mediators that might impair immunity. We investigated whether washed platelet and red cell transfusions could improve clinical outcomes in adult patients with acute leukemia.

**Methods:**

A pilot randomized trial of washed, leukoreduced ABO identical transfusions versus leukoreduced ABO identical transfusions was conducted in 43 adult patients with acute myeloid or lymphoid leukemia during 1991–94. Primary endpoints to be evaluated were platelet transfusion refractoriness, infectious and bleeding complications and overall survival.

**Results:**

There were no significant differences in infectious or major bleeding complications and only one patient required HLA matched platelet transfusions. Minor bleeding was more frequent in the washed, leukoreduced arm of the study. Confirmed transfusion reactions were more frequent in the leukoreduced arm of the study. Overall survival was superior in the washed arm of the study (40% versus 22% at 5 years), but this difference was not statistically significant (p = 0.36). A planned subset analysis of those ≤50 years of age found that those in the washed, leukoreduced arm (n = 12) had a 75% survival at five years compared with 30% in the leukoreduced arm (n = 10) (p = 0.037)

**Conclusion:**

This study provides the first evidence concerning the safety and efficacy of washed platelets, and also raises the possibility of improved survival. We speculate that transfusion of stored red cell and platelet supernatant may compromise treatment, particularly in younger patients with curable disease. Larger trials will be needed to assess this hypothesis.

## Background

In recent years, data have accumulated that platelet transfusion refractoriness and transfusion reactions in patients with hematologic malignancies can be reduced by use of leukoreduced [1-3] and/or ABO identical [4,5] platelet transfusions. Preliminary data also suggest that use of ABO identical [6] and leukoreduced transfusions [7] might potentially affect clinical outcomes such as survival and bacterial infection. Data also exist suggesting that alloimmunization to plasma antigens may play a role in platelet transfusion refractoriness, [8] and that removal of plasma supernatant can reduce the incidence of reactions to platelet transfusions [9]. One method for removing plasma supernatant from platelet concentrates is washing. However, washing involves loss of perhaps 20% of platelets. No clinical trial data exist comparing washed and unwashed platelet transfusions in terms of efficacy in preventing bleeding, and safety, in terms of unforeseen complications of transfusing platelets subjected to an additional manipulation.

Over the last two decades it has become apparent that allogeneic blood transfusions can modify host immunity and clinical outcomes [10]. Epidemiologic data, animal models, and, in some instances, randomized clinical trials demonstrate that transfusions reduce solid organ allograft rejection and repetitive spontaneous abortions, and increase the likelihood of post-operative bacterial infections [11]. Perhaps most controversial is the association between blood transfusion and cancer recurrence, which has been convincingly demonstrated in some animal models, [12] but for which randomized clinical trial evidence is lacking. We observed that patients with cancer had significantly greater recurrence rates if transfused with whole blood [13,14] rather than red cell concentrates, and this epidemiologic association has been confirmed by others [15].

Platelet transfusions are almost universally used in the supportive care of acute leukemia in adults. The original study design hypothesized a potential benefit from removing soluble immunomodulatory mediators in red cell and platelet concentrates derived from plasma and white cells. White cells and their secreted products are now largely removed prior to storage through filtration. However, there is now reason to be concerned about soluble platelet derived substances that would not be removed by pre-storage leukoreduction, as well as immunomodulatory mediators from plasma itself, such as IgG and soluble HLA antigens. More recent data document that stored platelet concentrate supernatants accumulate striking levels of biologic response modifiers during storage, including vascular endothelial growth factor (VEGF), soluble CD40L, histamine and transforming growth factor (TGF-β1) [16-18]. There is reason to believe that these molecules are infused at what may be clinically significant doses, and might alter recipient immune function. sCD40L has recently been demonstrated to be a growth promoting and apoptosis inhibiting factor for leukemic cells in vitro [19].

Platelet transfusions are given to patients undergoing myelotoxic chemotherapy for acute leukemia over a two to three week period when the peripheral blood immune system is regenerating. We speculated that platelet transfusions, in addition to causing platelet refractoriness and transfusion reactions, might impair anti-leukemic immunity because allogeneic transfusions have been shown to favor type 2 immunity (e.g., characterized by cytokines such as IL4 and IL10) and suppress type 1 cellular immunity (e.g., IL2, γ-interferon and TNF-α) [20,21]. There are data suggesting that host type 1 immunity may be important in the eradication of residual malignant cells after therapy [22].

To investigate the possible efficacy of washed platelet transfusions in preventing platelet refractoriness, transfusion reactions, bleeding and improving long term survival in adult acute leukemia, we performed a randomized trial in patients receiving either washed, leukoreduced, ABO identical platelet and red cell transfusions compared with our standard protocol of leukoreduced, ABO identical transfusions.

## Methods

### Patients

The diagnosis of acute leukemia was based upon laboratory results from our hematopathology laboratory. Only patients receiving chemotherapy with curative intent were included. Three patients died before receiving a full course of induction therapy but are included in the data. Data retrieval of clinical information was done in a blinded fashion. Patients and clinicians were not blinded as to study allocation due to the obvious difference in packaging of washed versus unwashed transfusions.

For acute myeloid or undifferentiated leukemia treatment invariably involved initial remission induction attempts with seven days of cytosine arabinoside and three days of an anthracycline. Patients with acute lymphoid leukemia received vincristine and prednisone, as well as other drugs. Patients with high risk features or those who failed to achieve remission received additional courses of induction therapy and/or additional agents depending on attending physician preference. Cytogenetic results, when available, were classified retrospectively according to a currently used scheme [23]. All stem cell transplants performed involved autologous bone marrow or that from an HLA identical sibling.

Patients entering remission also typically underwent consolidation therapy and bone marrow transplant depending on age, performance status, availability of a sibling HLA matched allogeneic donor and other factors. Our institutional review board for studies involving humans approved the study protocols and informed consent documents. Once placed on a particular transfusion protocol, as described below, a patient received only transfusions of that type throughout their treatment. This included consolidation, transplant and relapses, continuing until they were cured or died of their leukemia.

In 1991–94, 43 patients participated in a randomized trial of ABO identical, leukoreduced versus ABO identical, leukoreduced, plasma reduced (washed) transfusions. Study endpoints were platelet transfusion refractoriness, total platelet transfusion requirements during induction therapy, infections during induction therapy and overall survival. Randomization was arranged by our Department of Biostatistics employing computer generated random assignments and a blocked design with separate, sequential, sealed, opaque envelopes for patients with diagnoses of acute myeloid (AML) or acute lymphoid (ALL) leukemias. Leukoreduction for all patients was by post-storage, bedside filtration with Pall filters (RC50 and PL100), and plasma reduction of both red cell and platelet transfusions was by saline washing using the Cobe 2991 by a previously published method [24]. Neither transfusion service nor clinical staff was blinded to study assignment after opening of the envelope, but this was not considered essential as major clinical outcomes (platelet transfusion responsiveness and survival) were unlikely to be affected by staff knowledge of study assignment. Power calculations were not performed as the trial was considered primarily a feasibility trial. All adult patients with acute leukemia who were to receive full dose induction chemotherapy would be recruited during a three year period beginning in early 1991. A maximum of 60 patients were expected to be accrued.

Transfusion practice was consistent during the period of the study, employing almost exclusively whole blood derived random platelet concentrates, either ABO identical, leukoreduced, or washed, ABO identical, leukoreduced, according to protocol. Prophylactic transfusions were consistently given at morning platelet counts of <20 × 10^9^/l and usually prior to invasive procedures at <50 × 10^9^/l. Protocol violations in which patients received a transfusion of the incorrect type were <0.5% of all transfusions. All transfusions were treated with 2500 centigray gamma irradiation. We did not collect data on storage time of the red cells or platelets transfused in the study.

Patients were to be excluded from the analysis if they did not have acute leukemia or died prior to randomization. Since randomization occurred upon admission to the hospital with the diagnosis of suspected acute leukemia, some patients were found to have other diseases after randomization. These exclusions involved two patients who were randomized to the unwashed group but found not to have acute leukemia upon further investigation. Subgroup analysis was planned for patients ≤50 years of age because they are known to have substantially better prognosis, and for patients with acute myeloid leukemia, who may have a greater risk of HLA alloimmunization. Fifty years of age was also the maximal age for allogeneic bone marrow transplantation in patients with acute leukemia at the time of the study.

Data were collected by a blinded coauthor (JMH) from medical record review of the admission for initial induction, and follow-up data on survival obtained from the local tumor registry. The clinical data for blood component use and morbidity were collected only for the initial admission to achieve treatment time comparability. Bleeding was evaluated for the initial admission for remission induction, and defined as minor (1–2 days not requiring any therapeutic intervention such as wound site hematoma, guaiac positive stools, mild epistaxis not requiring transfusion nor packing, etc.) or major (requiring transfusion of red cells or otherwise mandating surgical or other therapeutic intervention). Refractoriness was evaluated for the entire duration of a patient's transfusion therapy until cessation of transfusions or death, and defined as the need for HLA matched platelet transfusions, as determined by the attending hematologist. Transfusion reactions were reported at the discretion of the nursing staff and evaluated by Transfusion Service residents who were not blinded as to the component received. A new fever or new rigors occurring during or shortly after a transfusion was considered a reaction whereas a fever or rigor that occurred shortly before the transfusion started was considered unrelated.

### Statistical methods

Statview 5.0 (SAS Institute, Cary, NC) was employed to calculate survival curves by the Kaplan-Meier method and Cox proportional hazards regressions. For continuous variables, the Mann-Whitney test was used for bivariate comparisons, and for categorical variables, Fisher's exact test or Chi square with continuity correction were employed as appropriate. No corrections were made for multiple comparisons, because except for the major outcome variables of survival, platelet refractoriness, infectious complications and platelet utilization, the data comparisons are considered exploratory.

## Results

**Figure 1:**
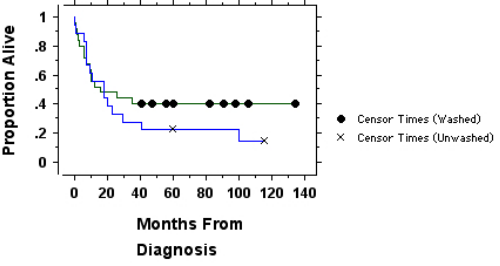
**Results of the randomized trial of washed (n = 25 patients) versus unwashed (n = 18) platelet transfusions**. There is no significant difference in survival by the logrank test (p = 0.36). Censored data points are patients remaining alive.

The demographic pre-treatment and initial hospitalization clinical outcomes data for patients in the study are shown in Tables 1 and 2, and the long term survival results are shown in the Kaplan-Meier plot in figure 1. Except for cytogenetics there were few differences between the two cohorts in terms of pre-treatment variables. Of those who had cytogenetics successfully determined there were more patients with poor risk cytogenetics in the washed arm (11 of 18; 61%) than in the unwashed arm (2 of 9; 22%) of the study (p = 0.11 by Fisher's exact test). There was no statistically significant increased need for red cell or platelet transfusions, nor increased bleeding in the patients receiving washed transfusions.

**Table 1:** Demographic and pre-treatment clinical variables according to type of transfusions given.

	ABO Matched, Leukoreduced	ABO Matched, Leukoreduced, Washed	P value by Mann Whitney or Fisher's Exact Test
N	18	25	
Age	48 ± 23	47 ± 17	0.89
Male	9 (50%)	11 (44%)	0.76
ALL/AML	4/14	5/20	0.99
Antecedent Hematologic Disorder (MDS)	0 of 18 (0%)	3 of 25 (12%)	0.25
Favorable Risk Cytogenetics	1 (6%)	0 (0%)	0.09*
Standard Risk Cytogenetics	6 (33%)	7 (28%)	
Poor Risk Cytogenetics	2 (11%)	11 (44%)	
Unknown Cytogenetics	9 (50%)	7 (28%)	
Admission Blast Count (×1000/μl)	25 ± 36	23 ± 41	0.59

All data are mean ± 1 SD. MDS = myelodysplastic syndrome

*For cytogenetics as a category overall

**Table 2:** Outcome variables according to type of transfusions received.

	ABO Matched, Leukoreduced	ABO Matched, Leukoreduced, Washed	P value by Mann Whitney or Fisher's Exact Test
N	18	25	
Red Cells (units)	16 ± 12	16 ± 6.2	0.21
Platelets (units)	84 ± 100	73 ± 49	0.64
Platelets/Day (units)	1.8 ± 1.2	1.9 ± 1.3	0.90
Red Cells/Day (units)	0.4 ± 0.1	0.4 ± 0.3	0.39
Platelets/Red Cell	4.6 ± 2.4	4.4 ± 2.5	0.74
Courses of Induction Chemotherapy	1.3 ± 0.6	1.3 ± 0.4	0.90
Length of stay (days)	43 ± 24	42 ± 17	0.47
Received HLA matched platelets	0 of 18 (0%)	1 of 25 (4%)	
Days with fever >37 degrees Celsius	16 ± 11	15 ± 9.2	0.90
Days of antibiotics	36 ± 26	36 ± 18	0.25
Positive Microbial Cultures	1.0 ± 1.2	1.3 ± 1.4	0.53
Days with bleeding	0.62 ± 2.2	0.58 ± 1.2	0.19
Reported Transfusion Reactions per Patient	0.4 ± 0.8	0.4 ± 0.7	0.91
Complete remission at discharge	12 of 18 (67%)	17 of 25 (68%)	1.00
Received BMT	8 of 18 (44%)	14 of 25 (56%)	0.54

All continuous variables results are means ± 1 SD.

Clinically evident bleeding was uncommon (8 of 43 patients, 19%) and major bleeding occurred in only 2 of 43 patients (5%). Minor bleeding was more common in those receiving washed platelets (6 of 25, 24%) than unwashed (0 of 18, 0%) (p = 0.03 by Fisher's exact test) but major bleeding occurred in only one patient in each arm of the study (1 of 25 versus 1 of 18; not significantly different). Bleeding was in all instances related to specific anatomic lesions, such as minor epistaxis, hematomas at the site of trauma or invasive procedures, or guaiac positive stools. For six patients these mild bleeding episodes were only seen on one or two days during their admission, without generalized petechiae or purpura. One patient in the washed arm had melena for five days, which resolved. The only life threatening bleeding was non-fatal and occurred in one patient in the unwashed arm of the study who experienced eight days of hemoptysis and received 416 units of platelets and 52 units of red cells during induction therapy. Bleeding did not influence overall survival at last follow-up, which was 38% in those who had bleeding and 35% in those patients with no bleeding (p = 0.94 by logrank test). Patients with bleeding required more platelet (mean of 141 ± 118) (1 SD) and red cell transfusions (21 ± 13) than patients with no bleeding (61 ± 44 platelets and 15 ± 7 red cells), but this was almost exclusively due to the one patient in the unwashed arm of the study with eight days of hemoptysis. When this patient was removed from the analysis, the patients with bleeding received no more transfusions than those without bleeding.

Reported numbers of transfusion reactions were similar in both arms of the study (a mean of 0.4 per patient). In the washed arm of the study there were eight transfusion reactions reported due to intercurrent fever and two allergic reactions (rash and/or urticaria) to unwashed platelet transfusions given in violation of protocol due to clinical urgency. In the unwashed arm of the study there were three transfusions with intercurrent fever and four allergic reactions. Reactions were reported in 7 of 25 (28%) of patients in the washed study arm and 7 of 18 (39%) of the patients in the unwashed arm (p = 0.52). When evaluated by the transfusion medicine resident, seven of the reactions in the washed arm were considered to be pre-existing fever and the reported reaction likely unrelated to transfusion. When only "on protocol" transfusions judged causally related by a blood bank physician are considered, the patients in the washed arm were less likely to experience reactions (1 of 25; 4%) than patients in the unwashed arm (7 of 18; 39%) (p = 0.0058 by Fisher's exact test). During the course of the treatment, three of 18 patients randomized and analyzed in the unwashed arm had severe or repeated transfusion reactions and were placed on a washed protocol for future transfusions.

As determined by a Cox proportional hazards regression, survival was not associated significantly with type of leukemia (p = 0.37), cytogenetic results (p = 0.63), or receipt of washed blood transfusions (p = 0.62) but was significantly associated with age (p = 0.0002), with younger patients surviving longer.

**Figure 2:**
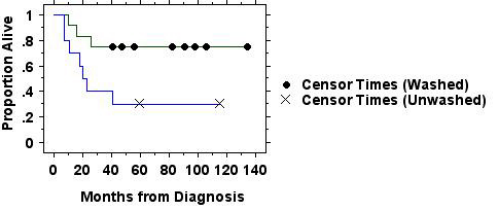
**Survival in those patients in the randomized trial of washed (n = 12) versus unwashed (n = 10) platelet transfusions ≤50 years of age is plotted by the Kaplan-Meier method. **Those in the washed group had significantly better survival (p = 0.037 by logrank test). Two of the patients in the washed group were both alive and in remission at last recorded follow-up of 47 months. Two of the patients in the unwashed arm were both alive and in remission at last recorded follow-up of 116 months. At the minimum follow-up time of 41 months 9 of 12 patients in the washed arm were alive and in remission, as compared with 3 of 10 in the unwashed arm.

Because long-term survival in acute leukemia is uncommon in those over the age of 50–60 years, we also performed a planned subset comparison of the 22 patients in the trial ≤50 years of age. We recognized that this subset would be small, rendering statistical analysis more difficult. These results are shown in figure 2, along with demographics on these patients in Tables 3 and 4.

**Table 3:** Demographic and pre-treatment clinical variables according to type of transfusions given in those ≤50 years of age.

	ABO Matched, Leukoreduced	ABO Matched, Leukoreduced, Washed	P value by Mann Whitney or Fisher's Exact Test
N	10	12	
Age	30 ± 9.4	32 ± 9.5	0.77
Male	5 (50%)	6 (50%)	1.00
ALL/AML	1/9	4/8	0.32
Antecedent Hematologic Disorder (MDS)	0 of 10 (0%)	0 of 12 (0%)	1.00
Favorable Risk Cytogenetics	0 (0%)	0 (0%)	0.09
Standard Risk Cytogenetics	5 (33%)	2 (28%)	
Poor Risk Cytogenetics	1 (11%)	6 (44%)	
Unknown Cytogenetics	4 (50%)	4 (28%)	
Admission Blast Count (×1000/μl)	29 ± 49	29 ± 49	0.56

All data are mean ± 1 SD. MDS = myelodysplastic syndrome

**Table 4:** Outcome variables according to type of transfusions received in those ≤50 years of age.

	ABO Matched, Leukoreduced	ABO Matched, Leukoreduced, Washed	P value by Mann Whitney or Fisher's Exact Test
N	10	12	
Red Cells (units)	20 ± 14	16 ± 6.2	0.93
Platelets (units)	127 ± 121	69 ± 52	0.23
Platelets/Day (units)	2.4 ± 1.1	1.4 ± 1.0	0.039
Red Cells/Day (units)	0.4 ± 0.06	0.4 ± 0.1	0.39
Platelets/Red Cell	5.9 ± 2.0	4.1 ± 3.0	0.069
Courses of Induction Chemotherapy	1.5 ± 0.8	1.2 ± 0.4	0.31
Length of stay (days)	50 ± 30	47 ± 18	0.56
Received HLA matched platelets	0 of 10 (0%)	0 of 12 (0%)	1.00
Days with fever >37 degrees Celsius	19 ± 12	14 ± 9.1	0.36
Days of antibiotics	44 ± 31	40 ± 21	0.74
Positive Microbial Cultures	1.1 ± 1.3	1.4 ± 1.5	0.76
Days with bleeding	1.0 ± 2.8	0.46 ± 0.67	0.36
Transfusion Reactions per Patient	0.4 ± 0.7	0.4 ± 0.5	0.73
Complete remission at discharge	9 of 10 (90%)	11 of 12 (92%)	1.00
Received BMT	8 of 10 (80%)	11 of 12 (92%)	0.57

All continuous variables results are means ± 1 SD.

There were more patients with ALL (p = 0.32) and more patients with poor risk cytogenetics in the washed arm of the study (p = 0.09). Otherwise there were few differences between the patients ≤50 years of age in the two cohorts. Patients in the washed arm had significantly better overall survival and required fewer platelets per day or per red cell transfused than patients in the unwashed arm. There were no significant differences in bleeding, use of growth factors or white cell transfusions between the two arms of the study, in either the entire or younger cohorts (data not shown but available from the authors on request).

Keeping in mind the extremely small number of patients involved, a Cox proportional hazards regression was performed on these younger patients in the randomized trial. Receipt of washed transfusions was a significant predictor of longer survival (p = 0.011), as was cytogenetics (patient's with unknown cytogenetics having poorer survival, p = 0.027) but age and type of leukemia were not statistically significant predictors of survival time.

**Figure 3:**
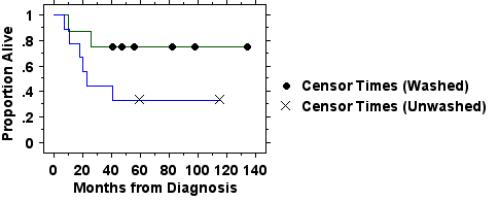
**Survival in those patients in the randomized trial of washed (n = 8) versus unwashed (n = 9) platelet transfusions ≤50 years of age with AML is plotted by the Kaplan-Meier method. **Those in the washed group experienced better survival but this was not statistically significant (p = 0.10 by logrank test). At a minimum follow-up of 41 months, 6 of 8 patients in the washed arm were in remission and alive compared with 3 of 9 in the unwashed arm (two of the patients in the unwashed arm were alive and in remission at 116 months).

The survival of all patients ≤50 restricted to those with AML is shown in Figure 3 confirming that the patients in the washed arm did not survive longer solely because of the larger number of patients with ALL in that arm of the study.

In the subset of all patients ≤50 years of age with either type of leukemia who both achieved a complete remission and also received definitive post-remission therapy in the form of bone marrow transplantation, the survival in patients receiving washed transfusions (n = 12) was significantly better than those receiving unwashed transfusions (n = 8) (p = 0.031 by log rank test) (graph not shown).

## Discussion and conclusions

To our knowledge, these data represent the first randomized trial of washed platelet transfusions in any setting. The issues of the efficacy and safety of washed platelet transfusions are of importance because (1) some patients require plasma-reduced transfusions to treat allergic or febrile reactions, (2) new methods of viral and bacterial pathogen-inactivation for transfusions may require washing prior to transfusion, and (3) a growing body of evidence suggests that immunologically important molecules are present in the stored supernatant of blood transfusions that might, speculatively, affect clinical outcomes. Our study design was less likely to detect a benefit of platelet washing because, unlike many centers, we routinely use ABO identical platelet transfusions for patients with leukemia. There is evidence from two small randomized trials in leukemia that this reduces refractoriness [4,5] and increases survival [6]. Preliminary results exist that ABO matching may also reduce morbidity and mortality in surgical patients [25].

There was no apparent benefit to washed transfusions in terms of reduced platelet transfusion refractoriness, reduced bacterial infections, reduction in reported febrile or allergic transfusion reactions or reduced length of stay. Confirmed transfusion reactions were less frequent in the washed arm of the study, and 15% of the patients in the leukoreduced arm of the study had severe or repeated reactions that led to their receiving washed transfusions, which abrogated those reactions. Our data do provide evidence, for the first time, that washed platelet transfusions are probably as safe and efficacious as standard leukoreduced transfusions. The mean number of days with bleeding was marginally but not significantly reduced in the patients in the washed group, as were the number of platelet transfusions needed per day in younger patients, and the ratio of platelet transfusions to red cell transfusions. However, there was more minor bleeding in the washed group raising the possibility that washed platelets are slightly less effective. However, the prevalence of major bleeding requiring treatment was the same in each group and very low (<5%). The only life threatening hemorrhage in these 43 patients occurred in a patient in the leukoreduced arm.

This study is also the first attempt to investigate whether transfusion practices during initial induction therapy are associated with changes in overall survival in acute leukemia in adults. The underlying rationale for studying this issue are observations demonstrating that allogeneic transfusions alter host T cell and natural killer cell immune function in surgical patients and experimental animals [11]. The major causes of death in adults with leukemia are failure to achieve complete remission, and relapse after achieving complete remission. There is some evidence from the allogeneic bone marrow transplant literature that host immune function may play a role in preventing relapse during the post-treatment period, but there are no data that demonstrate a role for the immune system in achieving complete initial remissions [26]. Our study found no difference in complete remission induction success rates with differing transfusion protocols, but does support the possibility of an association between type of red cell and platelet preparation transfused and long term survival.

Large numbers of red cell and platelet transfusions are given to patients with acute leukemia during the period of recovery from aplasia that is caused by cytotoxic chemotherapy given during remission induction. Additional transfusions are given during consolidation and bone marrow or peripheral blood stem cell transplantation. Many of these transfusions occur during the period when at least the bone marrow and peripheral blood compartments of the immune system are reconstituting and might be susceptible to the immunomodulatory effects of transfusions. Both white cell and platelet-derived mediators are present in the transfused red cells as well as platelets. Prestorage leukoreduction (not employed in this study) removes the vast majority of the white cells and platelets from stored red cells, and red cell concentrates would be expected to have much lesser concentrations of biologic mediators, even without washing. Platelet derived mediators would, of course, still be present in prestorage leukoreduced platelet concentrates.

Transfusions are known to cause suppression of type 1 cellular immunity and upregulation of type 2 humoral immunity [20,21]. Such immune deviation could, speculatively, impair host defenses provided by T cells, dendritic cells and natural killer cells that might be involved in the eradication or control of residual tumor. Potential mediators of down regulation of cellular immunity by transfusions include allogeneic white cells, red cells or platelets, ABO antigen-antibody complexes formed after repeated ABO non-identical platelet transfusions, and the stored supernatant of, in particular, platelet concentrates. Transfusion of stored platelet supernatant plasma might be hypothesized to mediate such effects by immunoregulatory and tumor growth promoting factors, such as sCD40L or angiogenic factors such as VEGF [16-18].

Leukoreduction filters remove white cells from the transfused blood components. In the case of pre-storage leukoreduction, filtration also removes some of the biologic response modifiers secreted by white cells during storage. Platelet concentrate supernatant contains large amounts of mediators such as soluble CD40L (CD154), VEGF, TGF-β1, histamine and other biologic response modifiers that might impair cellular immunity [16-18]. Washing immediately prior to transfusion removes most soluble materials from the transfused platelet and red cell transfusions, including those released during storage from the platelets, red cells and white cells. Our data provide initial support for the novel hypothesis that changing transfusion practices could play a role in long-term survival in acute leukemia, particularly in younger patients who can be potentially cured.

There are distinct limitations to what can be concluded from our data. The number of patients studied is very small and almost all of them had AML. The statistically significant improvement in survival in patients ≤50 years of age with AML who had unusually good survival represents a subset analysis, not a primary outcome group and thus may represent the play of chance. The survival of 75% of any cohort of adult patients with acute leukemia for 4–5 years is a distinctly unusual circumstance [27]. This could be due to the benefits of receiving washed transfusions, but could also be a chance occurrence in a small cohort of patients. Our overall survival in all patients of 40% in the larger cohort of patients of all ages and risk factors in the washed arm is equivalent to the best survival that has been reported in low risk, younger patients in other trials from the period of the early 1990s [27]. As shown in the figures, there was only one relapse and death that occurred at beyond five years in our cohorts, with a number of patients alive and in remission at 5–12 years after diagnosis. Thus it appears that if there was benefit from washing of transfusions, it probably involves an increased likelihood of durable remission, rather than purely delaying relapse.

These data, however promising, raise hypotheses for additional testing rather than proving a principle. If transfusion practice impacts anti-leukemic immunity, and/or survival as our data suggest, a moderate sized randomized clinical trial should be able to confirm this in relatively few years. This is a propitious time for such trials as the need for prophylactic platelet transfusion therapy is being revisited. The question is being raised as to whether platelet transfusions in non-bleeding patients are truly necessary or need be as frequent as currently employed in this disease [28]. One strategy for randomized trials would be to randomize patients to only therapeutic platelet transfusions (transfusion only for bleeding manifestations) versus current standard practice of prophylactic transfusions at a set threshold such as 10 × 10^9^/μl.

Platelet washing is time consuming, delaying transfusion by about 2.5–3 hours, may present an additional opportunity for bacterial contamination, and involves some loss of platelets (about 20%). Platelet washing may not be feasible in clinically urgent situations. Direct costs are modest, at less than $40 per transfusion. Washing adds about $500–2000 to the total cost of caring for patients with acute leukemia in our center from diagnosis to cure or death. This is less than 0.5–1% of the total costs of treating AML or ALL with curative intent. There are few or no proven side effects of platelet washing other than reduced dose of platelets transfused. There are some additional potential clinical benefits of washing including a reduced likelihood of transfusion complications such as febrile non-hemolytic transfusion reactions, transfusion-related acute lung injury, and allergic reactions. These preliminary data, albeit from a very small number of patients, raise the possibility that some patients with acute leukemia might benefit from washed transfusions. Larger studies are indicated to explore this possibility.

## Competing interests

Gambro BCT provided partial support for these studies. Dr. Blumberg has received lecture honoraria and a previous research grant from Pall Biomedical Corp. and lecture honoraria from Baxter.

## Authors' contributions

NB and JMH had the original idea for the study. All authors contributed to design of the study, drafting of the manuscript and all revisions. NB supervised the laboratory preparation of blood components for the study. JMR provided overall clinical supervision of the study and care for many of the patients. JMH collected the data and JMH and NB analyzed the data.

## Pre-publication history

The pre-publication history for this paper can be accessed here:


